# The Assessment of Fusion Following Sacroiliac Joint Fusion Surgery

**DOI:** 10.7759/cureus.1787

**Published:** 2017-10-20

**Authors:** Hamid Abbasi, John A Hipp

**Affiliations:** 1 Tristate Brain and Spine Institute; 2 Research, Medical Metrics

**Keywords:** sacroiliac joint fusion, radiographic assessment, method, grading

## Abstract

Sacroiliac joint fusions (SIJF) have been the subject of many research studies. The technical success of an SIJF is in part determined by whether osseous bridging occurs across the sacroiliac joint (SIJ). However, no validated SIJF assessment method has been described. Our objective was to document previously described SIJF assessment methods and define and validate a detailed assessment system for SIJF. Our results are only intended to establish computed tomography (CT)-based guidelines for SIJF to be used in a subsequent large clinical study to correlate them with clinical outcomes.

The SIJF literature was reviewed to document previous descriptions of SIJF assessments. A detailed system was then developed for assessing SIJF from CT exams. To provide data that can be used to address a range of research questions, the system included assessing bridging bone relative to the SIJ anatomy, bridging bone immediately adjacent to the threaded implants crossing the joint, as well as bridging bone close to but not immediately adjacent to the implants. The system was applied to assessing SIJF from thin-slice CT exams in 19 patients 12 months following surgery. Two experienced radiologists implemented the assessment system, and in the event of a disagreement, an adjudicator was used.

Most prior studies provide very little detail about how SIJF was assessed. Using the new assessment system, the agreement between the primary readers was substantial (0.67 using Gwet’s AC1 statistic). Bridging bone representing a fusion of the SIJ was identified in most patients both immediately adjacent to the threaded implants crossing the joint, as well as distant to the implants.

A detailed radiographic assessment system proved to be applicable to SIJF. The assessment system includes explicit language describing the location and extent of bridging bone across the SIJ. Standardization of the assessment of the SIJFs may allow for a more meaningful comparison of data between studies.

## Introduction

The sacroiliac joint (SIJ) is a recognized source of low back pain [1, 2]. When conservative therapy fails and diagnostic tests, such as provocative maneuvers and SIJ injections, confirm the SIJ is the source of symptoms, a fusion of the symptomatic joint can be an effective treatment [3]. While clinical outcomes following SIJ fusion (SIJF) are the most commonly reported result in peer-reviewed publications, an SIJF should, by definition, minimize motion between the ilium and the sacrum, based on the clinical hypothesis that motion across the joint is causing symptoms. There is currently no validated method for assessing SIJF status; validating a method was the goal of the current investigation.

Directly measuring motion between the ilium and sacrum is currently not practical in routine clinical practice. Muscle action and gravitational loads across the SIJ are complex, and the SIJ is heavily supported by ligament structures which limit or reduce motion to levels so low that a clinical test that can differentiate between a fused versus an unfused joint based on motion is not available in a clinical setting [4, 5]. In general, similar to the mechanism-of-action for a spine fusion, SIJF aims to stop motion by facilitating the formation of a continuous bone bridge such that the ilium and sacrum move as one. Identification of bridging bone between ilium and sacrum is therefore considered a fundamental approach to verifying the technical success of an SIJF.

Although investigators have described the characteristics of natural bridging that may occur as part of SIJ degeneration [6], the characteristics of bridging that may occur following an SIJF are poorly understood, in part for lack of a standardized assessment system. The purpose of the current study was to 1) compare SIJF assessment methods and results that have been reported in the peer-reviewed literature; 2) document radiographic characteristics of SIJF using minimally invasive threaded implants; 3) define and apply a new radiographic assessment system for SIJF. Standardizing SIJF assessment methods is essential to facilitate the future comparison of results across multiple studies. We, therefore, completed a small study to standardize and validate the SIJF assessment methodology. The methodology will be used in a larger study to investigate the clinical correlation to outcome.

## Materials and methods

**Figure 1:**
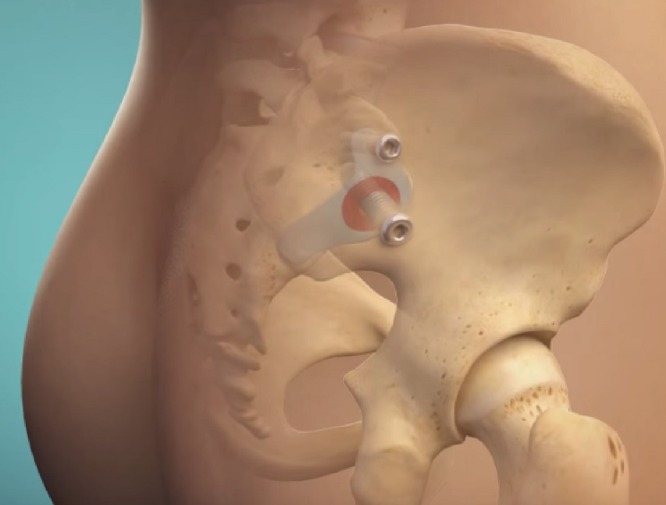
Rendering of the typical placement of the primary and secondary devices across the sacroiliac joint along with depiction of the area of the sacroiliac joint around the primary device that is decorticated and packed with bone graft during surgery.

SIJF with decortication and bone grafting had been performed in 19 patients with symptoms consistent with SIJ disorders that were unresponsive to conservative treatment. This study was approved by the New England Institutional Review Board (IRB) and the Mayo Clinic IRB and is registered with clinicaltrials.gov identifier NCT02425631. Patient inclusion criteria, the device, and the minimally invasive fusion procedure are described elsewhere in detail [7, 8]. Briefly, the procedure consisted of fluoroscopic identification of implant trajectories, use of a specially designed instrument for curettage and decortication of a region of the SIJ, bone grafting in the decorticated region, and implantation of a 12.5-mm diameter threaded primary and 6.5-mm diameter threaded secondary device across the SIJ (Figure 1). In five patients, only the primary device was implanted based on surgical considerations.

The SIJFs in the 19 patients were assessed via computed tomography (CT) exams obtained 12 months following the fusion operation. All CT exams were obtained using contiguous and non-overlapping 1-mm thick slices. Both bone and soft-tissue windows were available for most subjects, as well as sagittal and coronal plane reconstructions. Qualitatively, three independent radiologists with no relationship or contact with the manufacturer and blinded to clinical outcomes assessed each fusion. Two of the radiologists served as “primary” reviewers and read all of the imaging, and the third radiologist read the imaging to allow adjudication when there was a disagreement. The two primary radiologists were blinded to each other’s assessments. No intraobserver testing was performed. Prior to recording any assessments, the three reviewers studied a Microsoft PowerPoint-based training program that described the implants and the grading system. The training program was created by Medical Metrics, Inc. (Houston, TX), an independent imaging core lab.

**Figure 2:**
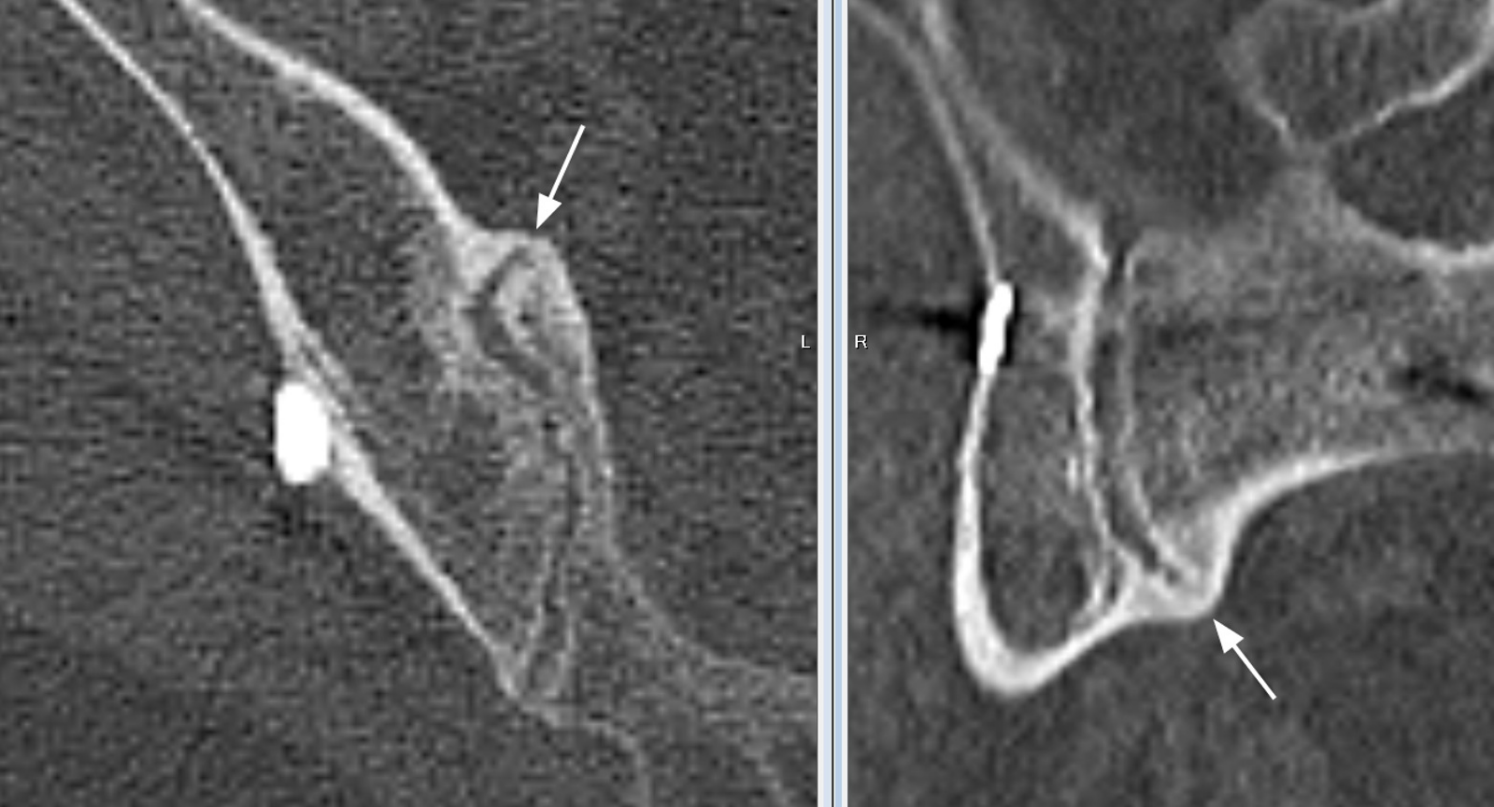
Axial and sagittal plane sections through a region of the sacroiliac joint where both primary readers identified solid bridging bone relative to the anatomy. Confirmation of the bridging bone seen at the intersection of two orthogonal planes was used to increase confidence in the assessments. The white arrows point to the bridging bone. Note that sections do not reproduce as well in print as they do on high-quality monitors.

**Figure 3:**
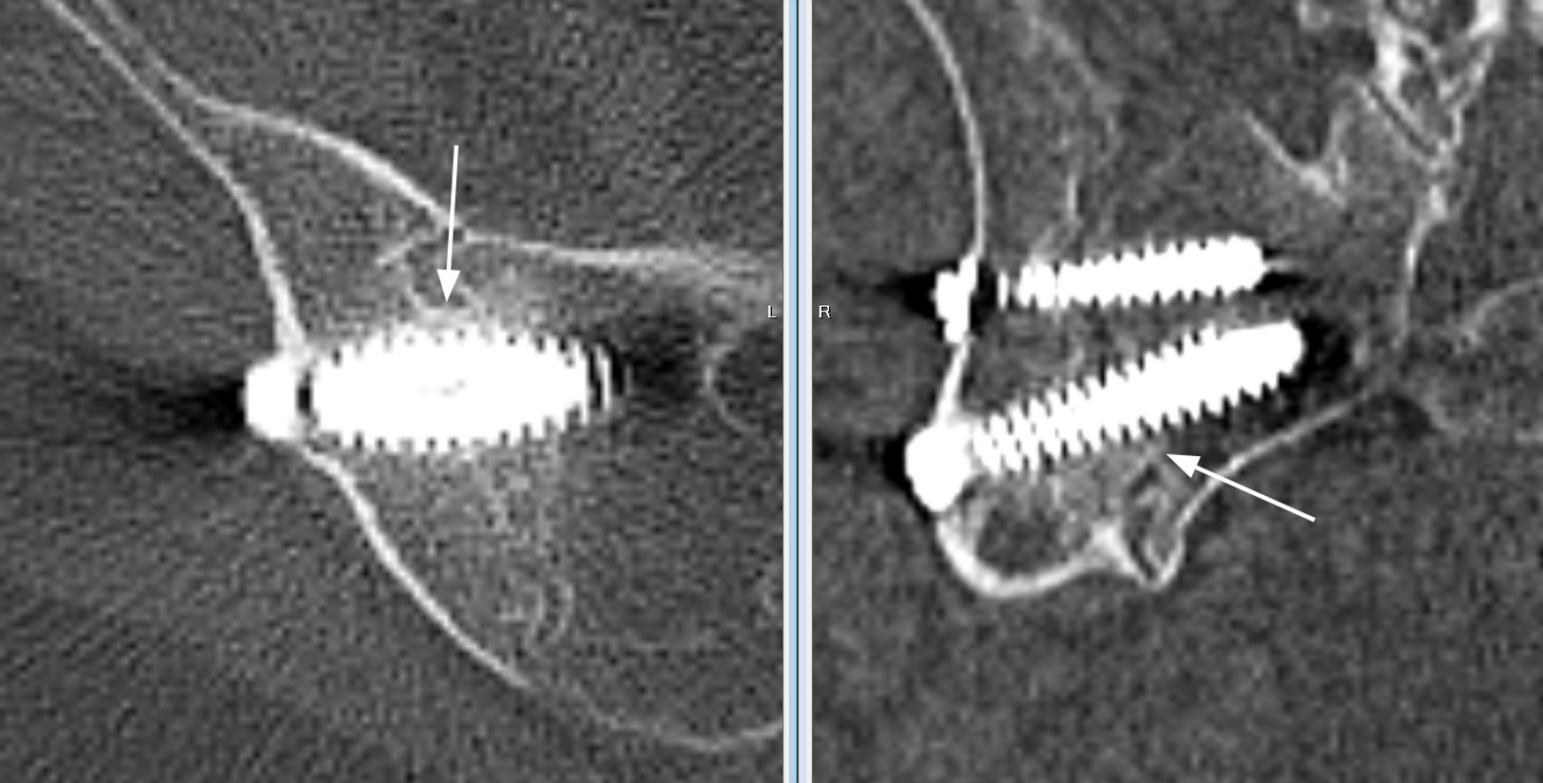
Axial and sagittal plane sections through a region of the sacroiliac joint where both primary readers identified solid bridging bone immediately adjacent to the primary device. Confirmation of the bridging bone seen at the intersection of two orthogonal planes was used to increase confidence in the assessments. The white arrows point to the bridging bone. Note that sections do not reproduce as well in print as they do on high-quality monitors.

The radiologists assessed fusion with respect to three references: 1) relative to the anatomy (Figure 2); 2) relative to the primary device (Figure 3); and 3) relative to the secondary device. They produce a limited metal artifact. The “relative to anatomy” assessment ignores the implants and captures any and all bridging bone and records whether the bridging was intra-articular or extra-articular. The “relative to the primary device” assessment captures the presence of bridging bone within the decorticated area, which extends 13.5 mm from the midline of the primary device, as well as whether the bridging bone was immediately adjacent to the primary device or outside the area of decortication. The “relative to the secondary device” assessment determined whether the bridging bone was immediately adjacent to the secondary device or more distal (but still related to the implant). In all three regions, the radiologists first recorded whether they observed evidence of bridging bone. If they observed evidence of bridging bone, they then graded the bone using a region-specific grading system. To justify classifying bridging as ‘solid,' a continuous path of bone must have been observed in two consecutive slices in a single plane (e.g., axial, sagittal or coronal) or continuous bone must have been observed at that location in two orthogonal views.

The region-specific grading systems for the subjective assessments are summarized in Tables 1-3. Each grading system was applied independently. Subjects with any bridging would have a bridging assessment performed using the grading system in Table 1. The radiologist also had the option of grading bridging as indeterminate (i.e., a reliable determination could not be made from the available imaging due to technical factors, suboptimal image quality, obscured anatomy, obstructed view or other imaging artifacts), or unable to assess (required images were missing or unavailable for review, or the relevant anatomy is not visible in the field of view). All radiographic analysis was performed by Medical Metrics, Inc.

**Table 1:** Grading system for fusion relative to the anatomy. This assessment was performed using axial, coronal, and sagittal slices of 1-mm thickness. If bridging is judged to be present, multiple qualifiers can be identified; e.g., solid extra-articular and possible intra-articular bridging may be recorded.

Grade	Description
Absent	No bridging bone
Present	Presence of solid or possible continuous bridging bone
Intra-articular (Solid)	Presence of solid continuous bridging across the treated joint within the joint space
Extra-articular (Solid)	Presence of solid continuous bridging across the treated joint outside of the joint space
Intra-articular (Possible)	Presence of possible continuous bridging across the treated joint within the joint space
Extra-articular (Possible)	Presence of possible continuous bridging across the treated joint outside of the joint space

 

**Table 2:** Grading system for fusion relative to the area of decortication and bone grafting surrounding the primary device.

Grade	Description
Absent	No bridging bone
Present	Presence of solid or possible continuous bridging bone
Adjacent (Solid)	Presence of solid continuous bridging within 13.5 mm of the center line of the primary device
Distant (Solid)	Presence of solid continuous bridging beyond 13.5 mm of the center line of the primary device
Adjacent (Possible)	Presence of possible continuous bridging within 13.5 mm of the center line of the primary device
Distant (Possible)	Presence of possible continuous bridging beyond 13.5 mm of the center line of the primary device

 

**Table 3:** Grading system for fusion relative to the secondary device.

Grade	Description
Absent	No bridging bone
Present	Presence of solid or possible continuous bridging bone
Adjacent (Solid)	Presence of solid continuous bridging within 8.5 mm of the center line of the secondary device
Distant (Solid)	Presence of solid continuous bridging beyond 8.5 mm of the center line of the secondary device
Adjacent (Possible)	Presence of possible continuous bridging within 8.5 mm of the center line of the secondary device
Distant (Possible)	Presence of possible continuous bridging beyond 8.5 mm of the center line of the secondary device

The level of agreement between reviewers on the presence/absence of bridging was assessed using Gwet’s AC1 statistic (Agreestat, Advanced Analytics, LLC, Gaithersburg, MD) because only a small number of subjects did not have bridging, and standard kappa statistics are not appropriate for this type of data distribution [9, 10]. An AC1 statistic of greater than 0.6 was considered the minimum acceptable agreement based on prior publications where CT exams were used to assess spine fusions [11-13]. The assessment of fusion from CT exams is subjective and confounded by volume averaging (even with nominally 1-mm slices) and beam hardening artifacts from the metal implants. Based on the peer-reviewed spine fusion literature (no prior CT-based SIJF studies were found where observer agreement was reported), an agreement of > 0.6 would be considered adequate.

Peer-reviewed literature was identified from 1) existing literature in the author’s files; 2) using multiple keyword searches conducted with Google Scholar and with PubMed; 3) by following citations to each of the identified papers using Google Scholar and other search engines; and 4) using the references cited in the literature that was identified through searches. These searches specifically targeted literature that described assessment of bone bridging across the SIJ. Review papers on SIJF were also used for content and references [14-16].

## Results

Assessment of fusion

Gwet’s AC1 chance-corrected agreement coefficient between the two primary reviewers was 0.67 for bridging relative to anatomy, 0.67 for bridging relative to the primary device, and 0.63 for bridging relative to the secondary device. Using the Landis and Koch criteria for judging kappa statistics, the agreement would be considered substantial [17]. Based on the adjudicated assessments, bridging bone was found in the SIJ in 79% of subjects, bridging bone was identified relative to the primary device in 79% of subjects, and bridging bone was identified relative to the secondary device in 71% of subjects (including only those subjects where a secondary device was implanted). When only bridging graded as solid (per the grading systems in Tables 1-3) is included, fusion was found relative to the anatomy in 74% of subjects. Solid or possible fusion was found immediately adjacent to the primary implant (within the area of decortication) in 13 subjects. Solid or possible fusion was found within 8.5 mm of the central axis of the 6.5-mm diameter secondary device in five subjects. With respect to the anatomy, solid or possible extra-articular fusion was observed in six subjects.

The publications that made any attempt to describe bridging or fusion have been organized into a publically accessible bibliographic database [18]. The complete and actual text describing the bridging or fusion assessment from each of these publications is provided in the notes field of the Zotero database.

## Discussion

Using the new grading system, evidence of bridging bone was observed in almost 80% of patients one year after percutaneous SIJF including decortication and bone grafting. This proportion of SIJF patients with bridging is in the range of the proportion of patients with evidence of fusion as reported in peer-reviewed literature. However, since few peer-reviewed papers provided anything more than superficial details about the fusion assessments and the reported methods were variable, fusion rates from the current study cannot be reliably compared to data from prior publications. Although it would have been valuable to be able to compare different SIJF assessment methods, there was no previously reported SIJF assessment method that was considered sufficiently well developed to justify use in a comparison study. Additionally, the low number of subjects in the current study did not allow for a comparison of radiographic fusion and clinical outcomes.

Metal artifact occurs immediately adjacent to the metal in some of the CT slices. This can confound the assessment of bone bridging. The radiologists did not report that the artifact prevented the application of the grading system in any of the subjects.

The focus of this study was the fusion assessment methodology. It was not intended to be a clinical outcomes study. A larger sample size would be needed to determine the association between fusion status and outcomes. To justify the radiation dose from a CT exam, it will be essential to validate that reliable assessment of fusion status is efficacious in optimizing clinical outcomes. However, that validation is not possible without first validating that the fusion status assessment is in itself reliable. That first step was the goal of the current study.

The literature regarding the meaning of fusion in the SIJ is incohesive. This paper attempts to validate a technique to establish radiographic fusion for use in subsequent studies to correlate with clinical outcomes. Many peer-reviewed publications describing the results of SIJF procedures do not report any systematic attempt to assess fusion status and focus primarily on patient outcomes (e.g., reduction in pain, the absence of adverse events, the absence of surgical re-interventions; e.g., Whang, et al. [19], Mason, et al. [20]). In those studies where bridging bone was assessed, a variety of approaches have been described for assessing the technical success of an SIJF but most publications provide no specific details.

Sachs, et al. commented in a paper describing their study of SIJFs that: “Radiological outcomes were not assessed; bony bridging cannot be reliably assessed on plain-film radiographs. Furthermore, in the absence of symptoms requiring further imaging, the cost and radiation exposure of CT scanning precludes such imaging studies from being performed routinely” [21]. A similar comment was included in a paper by Mason, et al. [20]. This rationale may have been implicitly used to justify no systematic fusion assessment in many published SIJF studies. Unfortunately, since most prior peer-reviewed publications describing SIJF studies do not apply a systematic assessment of bridging or indicate whether the fusion procedure minimized motion between ilium and sacrum, it remains unknown whether bone bridging across the joint influences outcomes or to what extent the surgery must minimize motion to achieve optimal outcomes. Variability in bridging or residual motion following SIJFs might explain a proportion of the variability in clinical outcomes, but this cannot be investigated without the systematic use of a validated grading system. It is also important to use a multi-reviewer independent assessment as observer agreement in fusion assessment can be poor [11, 22].

It is important to appreciate that fusion can occur across the SIJ in the absence of fusion surgery [23-26]. When this occurs, bridging is typically found in specific extra-articular locations [24, 25]. This suggests that intra-articular bridging is not a common natural mechanism for stabilization of the SIJ. Resnick and Resnik studied bridging osteophytes from autopsy studies and concluded that intra-articular bridging only occurs with ankylosing spondylitis [27]. Presumably, immobilization of the SIJ via fusion can speed up and establish extra-articular fusion where previous micro-motion prevented that from happening.

In some studies of implants that pass in a direct lateral trajectory across the SIJ, the investigators did not feel that it was necessary to have bone bridging between the sacrum and the ilium [28]. Instead, they felt that close apposition of bone to the implant on both the sacral and iliac side of the joint was sufficient. The assumption is that the rigid implants, if they are solidly anchored in both the sacrum and in the ilium, would effectively serve as a mechanical bridge that limits relative motion across the SIJ. In that scenario, no actual intra-articular or extra-articular bridging would be theoretically needed to stop motion between sacrum and ilium. Any bridging that does occur in that scenario would add to the stability. Since bone typically forms only where it is needed to support load-bearing requirements, it is possible that if robust rigid implants are solidly fixed in both the sacrum and ilium, then bridging bone would not form because it is not needed [29, 30]. In this scenario, verification of solid apposition of bone along the surfaces of the implants, in both the sacrum and ilium, would be more important than identification of bridging bone. The challenge with that assessment is the beam-hardening artifact that can occur in CT exams with substantial metal implants. In addition, the proportion of implant surface within the sacrum and the ilium that must have direct apposition with bone, and the required density of the apposing bone, remains unknown.

## Conclusions

Most peer-reviewed publications describing the results of SIJF procedures either do not attempt any assessment of bone bridging across the SIJ or provide only a minimal description of the fusion assessment. For this reason, no comparable reference data were found in the literature. A systematic assessment of bone bridging was completed one year after SIJF using a minimally invasive SIJF system in 19 patients. Evidence of fusion was observed in almost 80% of patients at 12 months. The bridging bone was frequently seen within the area that was decorticated and packed with bone graft across the SIJ. Standardization of SIJF assessment methods would facilitate a comparison of the results from multiple studies, including meta-analyses, and will also be required for individual practice performance metrics. A systematic and comprehensive assessment of bone bridging using grading systems defined a priori and implemented by independent radiologist reviewers, such as the methodology described in this study, could help to generate reliable data that determine whether SIJFs effect clinical outcomes and may facilitate comparisons between sites and between different SIJF surgical operations.
